# Supplementary material for: Screening of Membrane Protein Production by Comparison of Transient Expression in Insect and Mammalian Cells
Source: Biomolecules. 2023 May 11;13(5):817. doi: 10.3390/biom13050817 (PMC10216445; doi:10.3390/biom13050817)
Supplement: Supplementary file 1 [file biomolecules-13-00817-s001.zip › biomolecules-2188228-supplementary.pdf]

Supplementary data

Supplementary Note S1: Optimal MOI. The optimal MOI is related to the percentage of infection of insect cells. The optimal MOI is varying dependent on the method which has been used for the determination of the titre. Here we use the described end point method. Plaque assay, PCR analysis are other possible methods which may yield into a different estimation of the titre (pfu). For each method of determining the titre of the viral stock solution the optimal MOI has to be determined. Low MOI infection works well in Sf and ExpiSf cells because a superinfection will start after 48 hours once the first round of infection spreads new virus. Superinfection cannot be done in High Five cells. Therefore, the BEVS was not analysed in High Five cells for membrane protein expression screening.

Supplementary Note S2: Lentiviral transduction. Adherent HEK293S cells were transduced with lentiviruses as described in the materials and methods section at two temperatures (30°C and 37°C). Upon induction with 100 ng/ml doxycycline, GFP signals showed expression of the membrane protein (Figure 2). However, transduced cells could only be transferred to suspension cultivation in FreeStyleTM 293 or Expi293TM expression medium at relatively low (<3x 106 cells/mL) cell densities for the next 3 passages, making large-scale protein production in suspension cultures of lentiviral infected HEK293S cells less feasible.

Supplementary Note S3: Comparing the amounts of purified membrane protein. To compare the amounts of membrane target proteins (obtained after purification from different expression hosts), the GOI-3C-eGFP-His6 and GOI-3C-mCherry-Twin-Strep constructs were produced in 100 mL cultures in High Five cells. After 3 days of expression, the cell pellets were collected, disrupted by sonication and solubilized in 1% DDM followed by binding either to a Ni-NTA resin or Strep-Tactin resin and purification in presence of 0.05% DDM. Alternatively, the membrane proteins were extract after addition of 1:10 of a concentration of the DDM/CHS 10 x stock extraction buffer which contained 2% w/v cholesteryl hemisuccinate in 10% w/v DDM detergent solution. Hence the final concentration, added to the crude membranes to extract the membranes proteins, was 1% DDM+ 0.2% CHS (W/V), followed by further purification. Protein yields were compared by µg protein extracted from 100 mL of expression culture (see Table 4 and Figure 7B). Protein concentrations were determined from A280 absorbance measurements using a NanoDrop microvolume spectrophotometer (Thermo Fisher Scientific). The corresponding extinction coefficients and molecular masses were calculated from the protein sequences. However, as only an one-step purification was performed, the obtained data does not account for potential impurities of samples and different folding states of obtained proteins. For Ni-NTA purification all membrane fusion proteins were purified along with high host cellular impurities (Figure S4, Figure S5).

Supplementary Figure S1

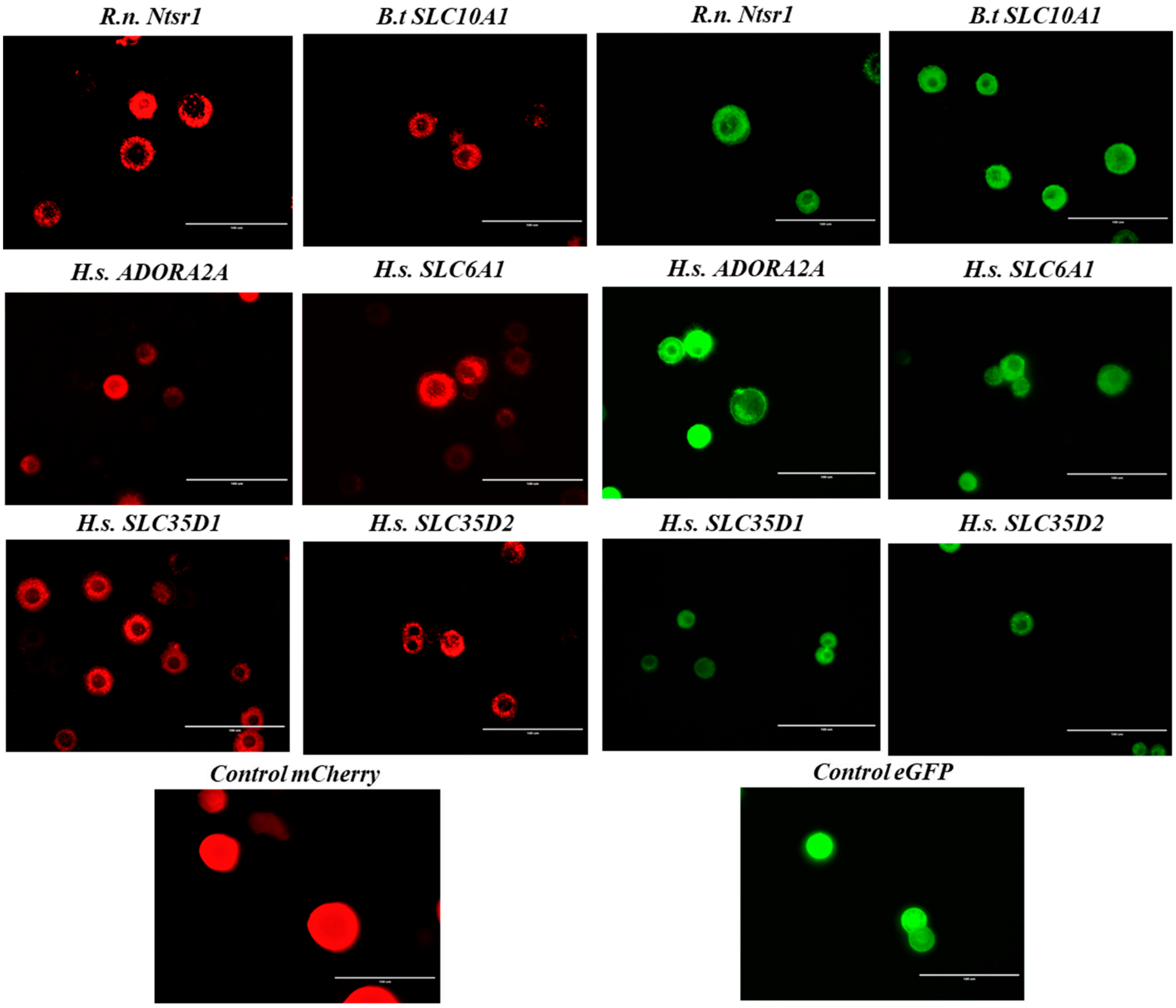

Supplementary Figure S1: Microscopic fluorescence analysis in High Five cells. Fluorescence micrographs were captured of the TGE using pOpiE2-GOI-3C-mCherry-Twin-Strep & pOpiE2-GOI-3C-eGFP-His6 expression constructs in insect High Five cells. Detection of fluorescent membrane fusion proteins in transient gene expression in High Five cells is presented at 48hrs post-transfection by EVOS fluorescence microscopy. Red fluorescence (Ex585nm, Em624nm) and green fluorescence (Ex485nm, Em530nm) were localized at either the cell wall or intracellular ER structures.

Supplementary Figure S2

A

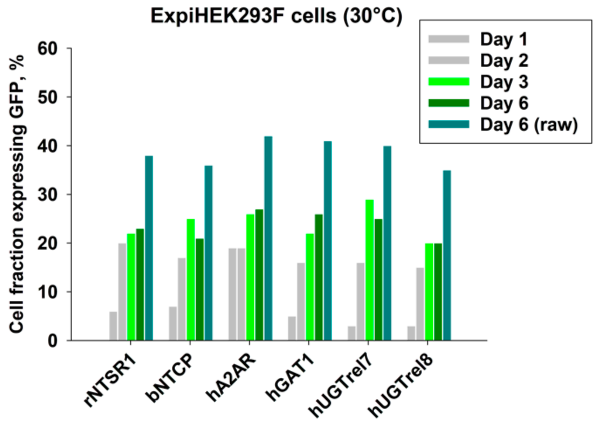

B

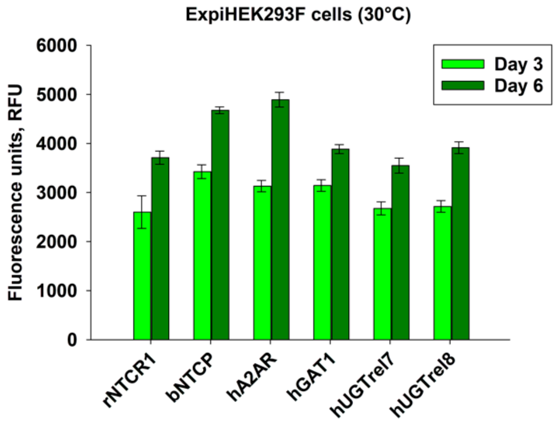

C

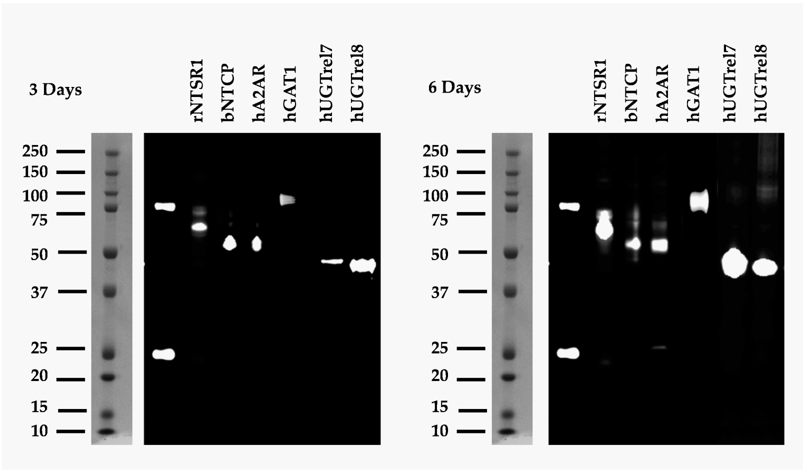

Supplementary Figure S2: Expression analysis of integral membrane proteins expressed using pOPINE vector in Expi293F cells at 30°C. (**A**) The efficiency of gene delivery for different targets (GOI-HRV3C-GFP6 constructs) at Day 1-6 post-transfection. The main GFP fluorescence signals are observed 48 hours post-transfection and after the addition of enhancers. The resulting efficiencies are plotted for both, the auto-fluorescence corrected data and the raw data at day 6. (**B**) In-cell GFP fluorescence signals for PEI-transfected Expi293F cells grown at 30°C. The maximal accumulation of GFP signals is observed on Day 6 post-transfection. The highest signals are observed for bNTCP and hA2AR targets. (**C**) SDS-PAGE In-Gel GFP fluorescence signals of membrane fusion proteins expressed in Expi293F cells at 30°C at two different time points (day 3 and day6). Higher GFP signals are observed for longer protein expression.

Supplementary Figure S3

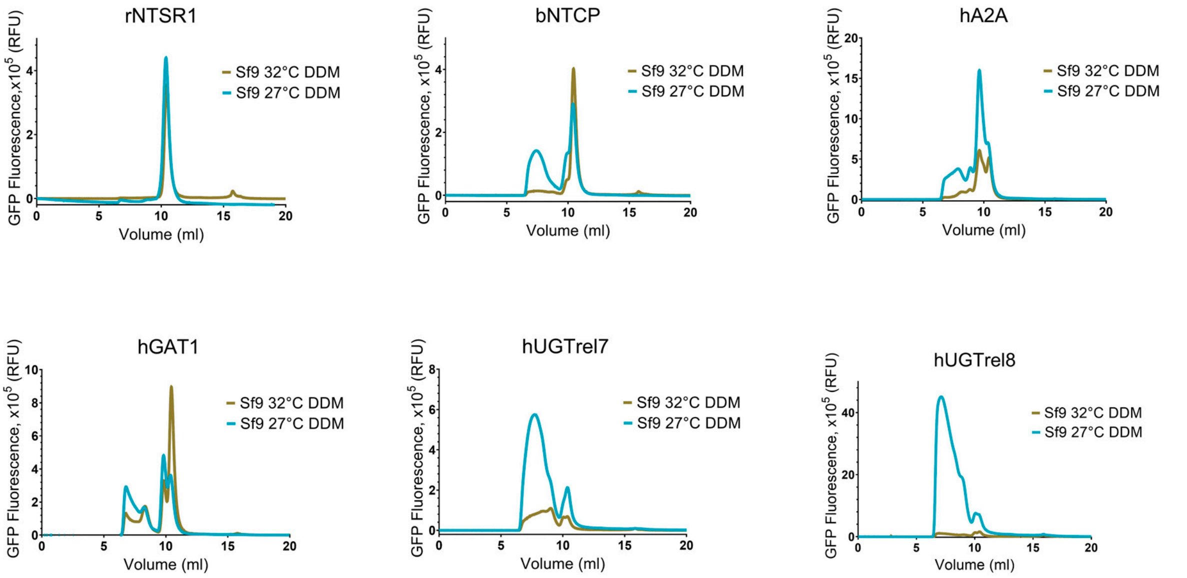

Supplementary Figure S3: Effect of temperature in Sf9 cells on oligomerisation state of targets. FSEC profiles of membrane fusion proteins produced in BEVS and purified in DDM. The purified samples expressed in ExpiSf cells extracted with DDM showed monodisperse monomers (rNTSR1, dNTCP, hGAT1 at 32°C) or polydisperse higher oligomers (dNTCP, hA2AR, hGAT1, hUGTrel7 and hUGtrel8 at 27°C) on FSEC column. A higher temperature of 32°C promoted overall a lower level of the production but the samples for bNTCP and hGAT1 showed better quality after purification with DDM by an increase in monodisperse material (sharper elution peak with fewer preceding shoulders).

Supplementary Figure S4

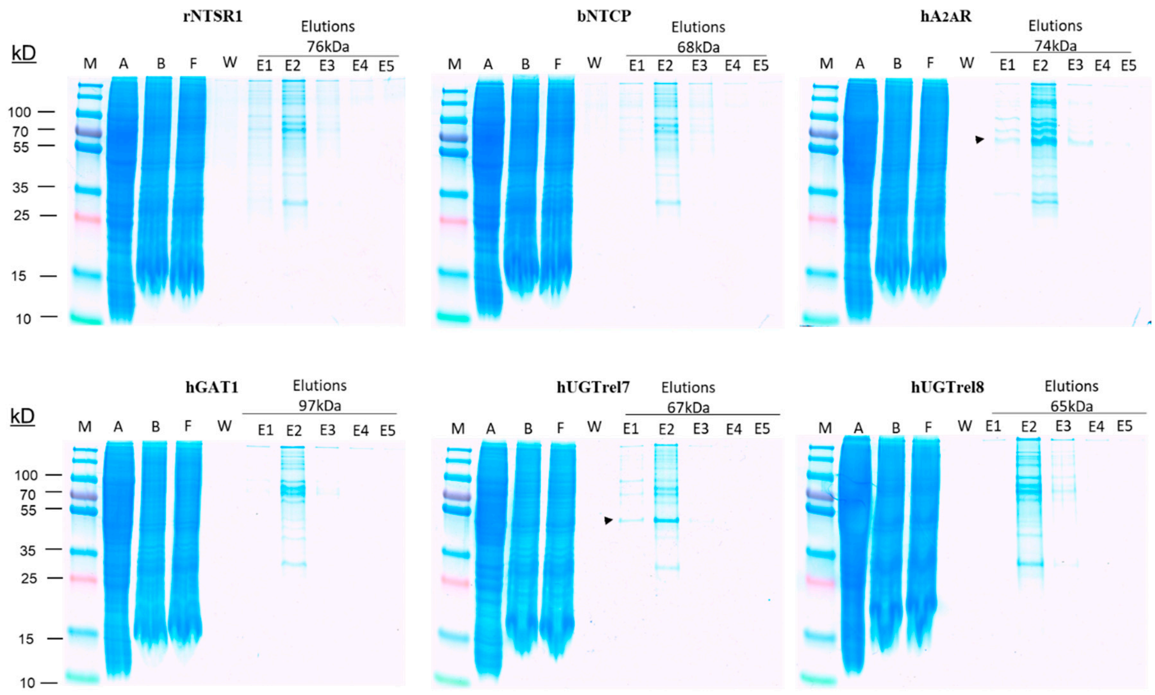

Supplementary Figure S4: Transient gene expression in High Five insect cells at 27°C by His-Tag purification. Six membrane fusion proteins were purified by His6-tag affinity purification using Ni-NTA resin in batch mode as described in the materials and method section. The expected size of the fusion protein is described above the lanes of the eluted fractions. The arrowhead indicates the positive visible band of the fusion protein. All other lanes show no clear positive signal compared to the background of co purified host proteins. (Where M=Molecular marker, A=Crude membranes, B=Detergent solubilized protein, F=Flow through, W=Wash, E(1-5)= Elution fractions 1-5).

Supplementary Figure S5

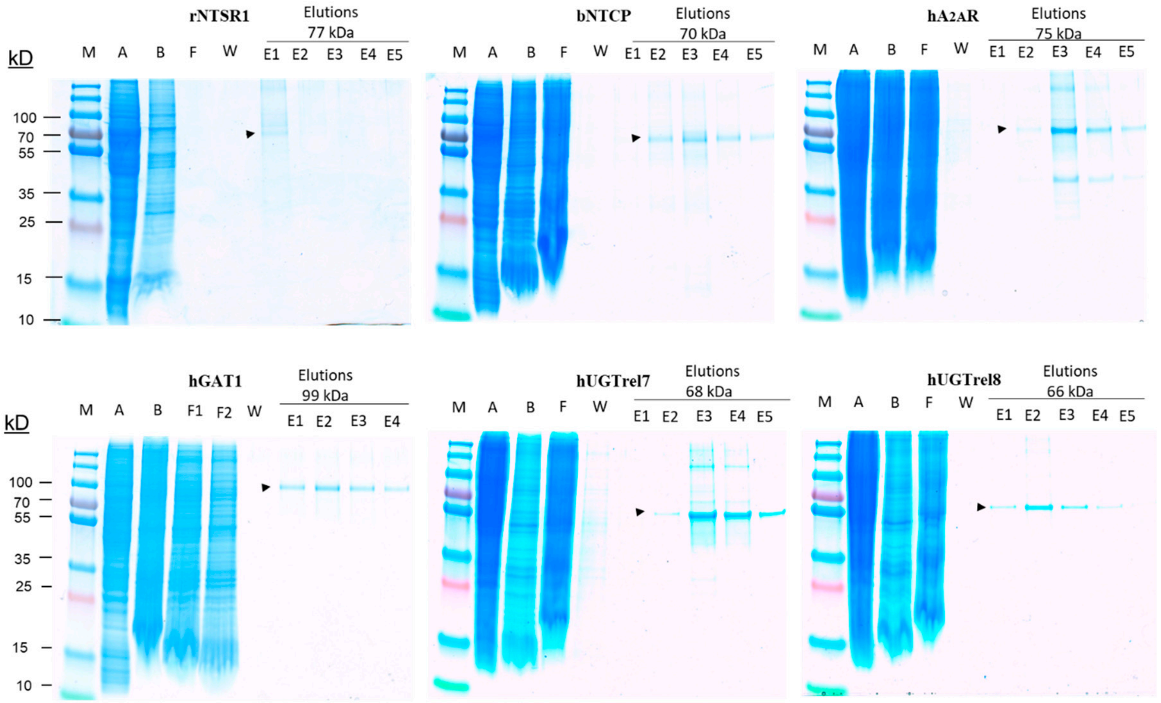

Supplementary Figure S5: Transient gene expression in High Five insect cells at 27°C by TwinStrep-Tag purification. Six membrane fusion proteins were purified by Twin-Strep-tag affinity purification using Strep-Tactin resin in batch mode as described in the materials and method section. The expected size of the fusion protein is described above the lanes of the eluted fractions. The arrowheads indicate the positive visible band of the fusion protein. Using the Strep-Tactin purification strategy showed good separation of the membrane fusion proteins from host proteins. (Where M=Molecular marker, A=Crude membranes, B=Detergent solubilized protein, F=Flow through, W=Wash, E(1-5)= Elution fractions 1-5).
